# A Retrospective Study on Vitamin D Status and Its Association With Cardiometabolic Risk Factors Among Children With Chronic Kidney Disease at King Abdulaziz University Hospital

**DOI:** 10.7759/cureus.39340

**Published:** 2023-05-22

**Authors:** Tala Fayoumi, Atheer Gari, Marah Alarawi, Samia Almutairi, Bashayer H Shalabi, Osama Safdar, Hanan Al Kadi

**Affiliations:** 1 Medical Intern, King Abdulaziz University Faculty of Medicine, Jeddah, SAU; 2 Medicine, King Abdulaziz University Faculty of Medicine, Jeddah, SAU; 3 Pediatrics Department, King Abdulaziz University Hospital, Jeddah, SAU; 4 Physiology Department, King Abdulaziz University Hospital, Jeddah, SAU

**Keywords:** pediatric nephrology, cardiometabolic risk factors, vitamin d, chronic kidney disease, cardiovascular disease

## Abstract

Background: Vitamin D deficiency is a significant global health issue. It is prevalent in chronic kidney disease (CKD) patients, which is an important cause of death among children. Many studies have found a link between low vitamin D status in CKD patients and cardiovascular disease (CVD) risk factors. However, there are no data on this relationship in children with CKD in Saudi Arabia.

Aims: We aimed to demonstrate this association among children with CKD admitted to the King Abdulaziz University Hospital (KAUH) in Jeddah, Saudi Arabia.

Materials and methods: Data were collected between June and August 2020 from a convenience sample of pediatric patients.

Results: In total, 153 pediatric patients with CKD stages 2-5 were admitted to the KAUH between 2010 and 2019, and 67.3% had CKD stage 5. Approximately 4.6% and 10.5% of the participants were overweight or obese, respectively. Patients who fell into the lower 25-hydroxyvitamin D (25[OH]D) tertile were older, had higher body mass index (BMI) values, and had higher blood pressure than those in the upper two tertiles; however, these differences were not statistically significant. There was a significant inverse association of 25(OH)D levels with BMI, blood pressure, and serum creatinine levels.

Conclusions: The results of this retrospective study suggest that patients with CKD and lower vitamin D levels have a higher BMI and blood pressure and are therefore at higher risk of developing CVD. Future prospective studies with a larger sample size are needed to confirm these findings. Randomized clinical trials are also needed to investigate the effect of sufficient vitamin D status on reducing CVD in patients with CKD.

## Introduction

Nearly one billion people around the world suffer from vitamin D deficiency [1]. Sun exposure causes the skin to synthesize vitamin D in the form of cholecalciferol, which then goes through two hydroxylation reactions as it reaches the bloodstream. The liver performs the first hydroxylation process, turning it into 25-hydroxyvitamin D (25[OH]D), while the second process takes place in the kidney’s proximal tubules, where it is transformed into 1,25-dihydroxyvitamin D (1,25(OH)2D) [2], which is the active, or hormone, form of vitamin D and is responsible for its biological functions [3].

Globally, renal dysfunction, often known as chronic kidney disease (CKD), is a significant public health issue with high rates of morbidity and mortality [4]. The most common causes in pediatric patients are congenital kidney and urinary tract abnormalities, followed by glomerulonephritis and inherited nephropathies [5]. Vitamin D deficiency, described as a 25[OH]D level <20 ng/mL, occurs frequently in people with CKD and increases the risk of morbidity and mortality [6]. Patients with CKD are exposed to age-related and other risk factors for 25(OH)D deficiency, including female sex, adiposity, proteinuria, low physical activity, peritoneal dialysis, diabetes mellitus, and reduced vitamin D receptor (VDR) [7].

Cardiometabolic disorders are a major contributor to cardiovascular disease (CVD), which is one of the leading causes of death for people with CKD [7]. The interaction between cardiovascular and metabolic risk factors, such as the association of CKD with obesity, hypertension, dyslipidemia, and insulin resistance, presents difficulty in terms of risk classification. Consequently, optimizing metabolic health is essential for managing children with CKD [8]. Vitamin D plays an important role in cardiovascular health [9], for example, in blood pressure regulation and heart, smooth muscle, and endothelial cell functions. Additionally, it has been discovered that there is a link between CVD and vitamin D in CKD patients.

Previous studies have found that low vitamin D levels in people with CKD are reliable indicators of disease development and mortality [10]. In a randomized clinical trial, high-dose cholecalciferol (2000-8000 IU/day) administration in children with CKD showed favorable effects on cardiovascular and endothelial parameters [11]. Another study in the US found that approximately 30% of deaths in patients with CKD were secondary to cardiovascular causes. Moreover, increased left ventricular mass and diastolic dysfunction have been linked to vitamin D deficiency [12]. Other studies have shown that children with CKD have a high prevalence of CVD risk factors that persist even after renal transplantation [13].

Therefore, understanding the effects of vitamin D deficiency on CVD development in pediatric age groups is of great interest. However, in Saudi Arabia, there are no data on the relationship between cardiometabolic risk factors and low vitamin D levels in children with CKD. Identifying cardiometabolic risk factors among CKD children with severe vitamin D deficiency would provide a basis for future research on the advantages of vitamin D supplementation and improving vitamin D status in lowering their cardiovascular risk. Thus, this study aimed to determine the association between cardiovascular and metabolic risk factors and low vitamin D levels in children with CKD in Jeddah, Saudi Arabia.

## Materials and methods

Study design and setting

This retrospective record review study was carried out between June and August 2020 at the Pediatric Nephrology Center of Excellence (PNCE) at King Abdulaziz University Hospital (KAUH), a tertiary center in Jeddah, Saudi Arabia. Ethical approval was obtained from the institutional review board of KAUH (Reference No. 366-20). This study was performed according to the ethical standards of the institutional and/or national research committee, the 1964 Helsinki Declaration, its later amendments, or comparable ethical standards. Informed consent was obtained from the parents or guardians of the children.

Study population

All pediatric patients of both sexes (aged 1-18 years) with CKD stage based on an estimated glomerular filtration rate (eGFR) <90 mL/min/1.73 m^2^ between 2010 and 2019 were included in the study. Children with congenital heart disease, diabetes, thyroid malfunction, renal cancer, those who underwent a renal transplant, and those with missing data on 25(OH)D levels were excluded.

Data collection instruments

Records were reviewed from the Phoenix system at KAUH using a data collection sheet via Google Forms. The data sheet included information on the patient’s demographics (date of birth, sex, and nationality), socioeconomic data (birth order, monthly income, and parents’ education), and family history of renal disease acquired by contacting a family member who knew the most about the child. Using the recorded height and weight, the body mass index (BMI) was calculated by dividing the weight in kilograms by the square of the height in meters. Heart rate, systolic blood pressure (SBP), and diastolic blood pressure (DBP) were also noted. Laboratory readings (eGFR mL/min/1.73 m^2^, serum 25(OH)D in nmol/L, serum creatinine in µmol/L, hemoglobin in g/dL, the parathyroid hormone in pmol/L, serum calcium in mmol/L, serum phosphate in mmol/L, albumin in g/L, and magnesium in mmol/L levels) were also recorded.

Risk factors were defined as follows

SBP and DBP above the 95th percentile for the child’s age and sex were considered to indicate high blood pressure. Obesity was defined as BMI ≥95th percentile for the child’s age and sex based on the Centers for Disease Control and Prevention’s growth charts, hypoalbuminemia as a plasma albumin level <30 g/L, hyperparathyroidism as a serum parathyroid hormone level >7 pmol/L, anemia as a hemoglobin level <10 g/dL, hyperphosphatemia as plasma inorganic phosphate >1.6 mmol/L, and hypercalcemia as plasma total calcium >2.52 mmol/L. The corrected calcium level (mmol/L) was calculated using the adjusted Payne formula [14]: serum calcium (mmol/L) + 0.02 * (40 - serum albumin [g/L]).

Data analysis

Statistical analyses were performed using Statistical Package for the Social Sciences (SPSS) version 21 (SPSS, version 23, IBM Corp., Chicago, IL, USA). The normality of the data was examined using the Shapiro-Wilk test. The terms "median" and "interquartile range (IQR)" were used to describe non-normally distributed data. For categorical variables, numbers and percentages were used, whereas the mean ± standard deviation (SD) was computed for continuous normally distributed variables. Tertiles of 25(OH)D were evaluated, and variables in the lower tertile were compared with those in the upper two. The differences between continuous and categorical variables were assessed using the Student’s t-test and the Chi-square test, respectively. The independent relationship between 25(OH)D level and various cardiometabolic risk factors was investigated using multiple linear regression analysis, which was adjusted for age, sex, nationality, eGFR, monthly income, mother's education, parathyroid hormone level, heart rate, corrected calcium level, and BMI. The threshold for significance was set at P<0.05.

## Results

In total, 153 pediatric patients from 1 to 18 years of age with CKD stages 2-5 (62% males) were admitted to KAUH between 2010 and 2019. More than two-thirds (67.3%) of the recruited children had CKD stage 5. The sociodemographic and clinical features of the patients are shown in Table 1. Approximately 95% of the patients were on vitamin D3 supplements; however, 44% had 25(OH)D levels of ≤50 nmol/L and 96% had high parathyroid hormone levels. Finally, 4.6% and 10.5% of the participants were overweight or obese, respectively.

**Table 1:** Characteristics of the study population (n=153) BMI: body mass index; 25(OH)D: 25 hydroxyvitamin D; CKD: chronic kidney disease; PTH: parathyroid hormone; Ph: serum phosphate.

Variables	Frequency (%)
Sex	
Male	95(62.1%)
Female	58(37.9%)
Age (years)	
1–5	52(34.0%)
6–10	54(35.3 %)
11–15	39(25.5%)
16–18	8(5.2%)
Nationality	
Saudi	88(57.5%)
Non-Saudi	65(42.5%)
Birth order	
1	13 (15.5%)
2	25(29.8%)
3	12(14.3%)
>3	34(40.6%)
Socioeconomic status	
Low <5000	43(52.4%)
Medium 5000–10000	23(28.0%)
High >10,000	16(19.5%)
Father education	
Not educated	10(12.0%)
Elementary	15(18.1%)
Secondary	26(31.3%)
Academic	32(38.6%)
Mother education	
Not educated	18(21.7%)
Elementary	7(8.4%)
Secondary	29(34.9%)
Academic	29(34.9%)
Family history of renal disease	
Yes	16(19.5%)
No	66(80.5%)
On vitamin D supplement	
Yes	146(95.4%)
No	7(4.6%)
25(OH)D (nmol/L)	
<12.5	9(5.9%)
12.5–25	15(9.8%)
25.1–50	43(28.1%)
50.1–75	33(21.6%)
>75	53(34.6%)
CKD stage	
Stage 2	8(5.2%)
Stage 3	14(9.2%)
Stage 4	28(18.3%)
Stage 5	103(67.3%)
BMI (kg/m^2^)	
Underweight	47(30.7%)
Healthy weight	83(54.2%)
Overweight	7(4.6%)
Obese	16(10.5%)
Blood pressure (mmHg)	
Hypertensive	79(55.6%)
Normal	63(44.4%)
PTH (pmol/L)	
Normal	6(3.9%)
Hyperparathyroidism	147 (96.1%)
Ph (mmol/L)	
Normal	61(39.9%)
Hyperphosphatemia	92(60.1%)

The study cohort's initial characteristics are shown in Table 2 for reference. The study cohort's mean 25(OH)D level (SD) was 65 (42) nmol/L.

**Table 2:** Baseline characteristics of the study population BMI: body mass index; eGFR: estimated glomerular filtration rate; IQR: interquartile range.

Variables	Mean ±SD	Median (IQR)
Age (years)	7.7±4.4	8.0 (4.0–11.0)
BMI (kg/m^2^)	16.0±5.6	15.0 (13.1–17.7)
Systolic blood pressure (mmHg)	121.8±23.1	119.0 (108.0–130.0)
Diastolic blood pressure (mmHg)	75.4±19.2	73.0 (62.0–86.0)
Hemoglobin level (g/L)	9.3±2.2	-
Vitamin D (nmol/L)	65.3±41.6	56.5 (33.1–90.4)
Parathyroid hormone(pmol/L)	84.7±72.8	59.4 (26.1–127.0)
Corrected calcium (mmol/L)	2.25±0.34	2.30 (2.08–2.48)
Serum phosphate (mmol/L)	1.97±0.85	1.76 (1.42–2.32)
Albumin (g/L)	30.4±7.4	
Serum creatinine (µmol/L)	509.1±369.5	430 (200.3–732.0)
eGFR (mL/min/1.73 m^2^)	16.3±16.8	9.8 (6.3–19.8)

Table 3 shows the different variables studied according to the tertiles of serum 25(OH)D levels. Patients in the lower 25(OH)D tertile were older and had higher BMI and blood pressure (both SBP and DBP) than those in the upper two tertiles; however, these differences lacked statistical significance. The relationships between the various examined variables and serum 25(OH)D levels are shown in Table 4. BMI, DBP, and serum creatinine levels showed statistically significant inverse correlations with 25(OH)D levels. In contrast, significantly higher 25(OH)D levels were positively associated with corrected calcium levels, albumin levels, and eGFR.

**Table 3:** Characteristics of the study population according to vitamin D tertiles BMI: body mass index; 25(OH)D: 25 hydroxyvitamin D; eGFR: estimated glomerular filtration rate.

Variables	Lower vitamin D tertile 25(OH)D ≤ 41.9 nmol/L (n=51)	Upper 2 vitamin D tertiles 25(OH)D > 41.9 nmol/L (n=102)	P-value
Age (years)	8.6±4.3	7.2±4.5	0.08
BMI (kg/m^2^)	17.3±6.2	15.3±5.2	0.05
Systolic blood pressure (mmHg)	122.3±23.7	121.6±22.9	0.86
Diastolic blood pressure (mmHg)	75.7±17.1	75.2±20.2	0.89
Hemoglobin level (g/dl)	9.3±2.5	9.2± 2.0	0.97
25(OH)D (nmol/L)	25.1±10.3	85.3±36.4	0.00
Parathyroid hormone (pmol/L)	95.0±73.8	79.5 ±72.2	0.23
Corrected calcium (mmol/L)	2.18±0.40	2.29±0.31	0.10
Serum phosphate (mmol/L)	1.97±0.95	1.97±0.79	0.99
Albumin (g/L)	28.9±8.3	31.2±6.9	0.07
Serum creatinine (µmol/L)	565.2±326.7	481.0±387.7	0.19
eGFR (mL/min/1.73 m^2^)	16.1±19.5	16.4±15.3	0.92

**Table 4:** Spearman’s correlation of baseline characteristics with 25(OH)D levels Values in the table are Spearman’s rho coefficients. **Correlation is significant at the 0.01 level. *Correlation is significant at the 0.05 level. BMI: body mass index; 25(OH)D: 25 hydroxyvitamin D; SPB: systolic blood pressure; DBP: diastolic blood pressure; HB: hemoglobin; PTH: parathyroid hormone; C-Ca: corrected total serum calcium; iPh: serum phosphate; Ca-Ph: calcium phosphate product; GFR− glomerular filtration rate.

	25(OH)D	Age	BMI	SBP	DBP	HB	PTH	C-Ca	PH	Ca-Ph	Albumin	Creatinine	GFR	CKD
25(OH)D	1.000	−0.226**	−0.219**	−0.145	−0.231**	0.072	−0.155	0.168*	0.030	0.084	0.198*	−0.228**	0.165*	−0.123
Age	−0.226**	1.000	0.341**	0.347**	0.340**	0.152	−0.042	−0.109	−0.239**	−0.272**	0.058	0.203*	0.033	−0.031
BMI	−0.210**	0.341**	1.000	0.101	0.068	0.195*	0.006	−0.126	−0.110	−0.192*	0.099	0.027	0.083	−0.117
SBP	−0.145	0.347**	0.101	1.000	0.783**	−0.075	−0.008	−0.134	−0.122	−0.151	−0.133	0.284**	−0.152	0.163*
DBP	−0.231**	0.340**	0.068	0.783**	1.000	−0.085	0.077	−0.129	−0.149	−0.169*	−0.126	0.283**	0.160*	0.179*
HB	0.072	0.152	0.195*	−0.075	−0.085	1.000	−0.209*	0.220**	−0.220**	−0.130	0.202*	−0.346**	0.408**	0.468**
PTH	−0.155	−0.042	0.006	−0.008	0.077	−0.209*	1.000	−0.274**	0.258**	0.144	0.048	0.398**	−0.408**	0.468**
C-Ca	0.168*	−0.109	−0.126	−0.134	−0.129	0.220**	−0.274**	1.000	−0.140	0.207*	−0.190*	−0.156	0.176*	−0.094
iPH	0.030	−0.239	−0.110	−0.122	−0.149	−0.220	0.258	−0.140	1.000	0.915	−0.127	0.258	−0.354	0.326
Ca-Ph	0.084	−0.272	−0.192	−0.0151	−0.169	−0.130	0.144	0.207	0.915	1.000	−0.228	0.212	−0.288	0.291
Albumin	0.198	0.058	0.099	−0.133	−0.126	0.202	0.048	−0.190	−127	−0.228	1.000	−0.163	0.184	−0.195
Creatinine	−0.228	0.203	0.027	0.284	0.283	−0.346	0.398	−0.156	0.258	0.212	−0.263	1.000	−0.921	0.804
GFR	0.165	0.033	0.083	−0.152	−0.160	0.424	−0.408	0.176	−0.354	−0.288	0.184	−0.921	1.000	−0.829
CKD stage	−0.123	−0.031	−0.117	0.163	0.179	−0.428	0.468	−0.094	0.326	0.291	−0.195	0.804	−0.829	1.000

Table 5 shows the independent predictors of the studied cardiometabolic risk factors (adjusting for age, sex, nationality, eGFR, monthly income, mother’s education, parathyroid hormone level, heart rate, corrected calcium level, and BMI). Among the different cardiometabolic risk factors studied, 25(OH)D level was the only significant predictor of BMI and blood pressure (both SBP and DBP); BMI was reduced by 0.024 kg/m^2^ for every 1 nmol/L increase in 25(OH)D, while SBP decreased by 0.155 mmHg and DBP decreased by 0.130 mmHg.

**Table 5:** Independent predictors of the studied cardiometabolic risk factors BP: blood pressure; BMI: body mass index; iPH: inorganic phosphate; C-Ca: corrected total serum calcium.

Dependent variables	Predictors	β estimate (95% CI)	P-value
BMI (kg/m^2^)	Age	0.352 (0.147 to 0.557)	0.001
	25(OH)D	−0.024(−0.047 to −0.001)	0.038
Systolic BP (mmHg)	25(OH)D	−0.155 (−0.289 to −0.021)	0.024
Diastolic BP (mmHg)	25(OH)D	−0.130 (−0.241 to −0.019)	0.022

## Discussion

According to our findings, patients in the lower 25(OH)D tertile were older, had a higher BMI, and had higher blood pressure (both SBP and DBP) than those in the upper two tertiles; these differences were not statistically significant; however, according to regression analysis, the 25(OH)D level was found to be a significant predictor of BMI and blood pressure (both SBP and DBP).

These results are consistent with those of previous studies [14]. Furthermore, other studies have reported inconsistencies in the association between vitamin D levels and metabolic syndromes. However, they found that low vitamin D levels were associated with a higher risk of developing type 2 diabetes, as vitamin D helps to improve sensitivity to insulin [15-17]. Additionally, several studies have discovered that higher 25(OH)D levels are associated with a reduced risk of cardiovascular illnesses [18]. The differences between these findings could be due to different study types: studies that found positive correlations were observational studies, in which the patients were observed and outcomes measured to achieve more accuracy. Another reason could be that the patient’s data were poorly documented in the hospital’s system, resulting in false statistics.

Vitamin D and obesity

Our findings support other research findings, indicating that obese children and adolescents are more likely to be vitamin D deficient than their non-obese counterparts [19-22]. To explain this, several theories have been put forth on the links between vitamin D deficiency and obesity. First, because of societal stigma, obese people typically avoid going out in the daytime, hardly engage in any outdoor activities, and/or dress in clothing that covers most of their bodies, restricting sun exposure and cutaneous vitamin D production [23]. Alternatively, the increased local utilization of 25(OH)D could be explained by the presence of the enzyme 1-hydroxylase, which activates vitamin D in the adipose cells of obese people [23]. According to one study [24], regardless of the quantity of cutaneous vitamin D precursor present, the increase in serum 25(OH)D concentration was lower in obese persons after exposure to sunlight than in other individuals.

However, in line with earlier findings, evidence of a link between low vitamin D levels and obesity remains unknown. The majority of these results depend on variations in the VDR gene, which has been linked to obesity features in some studies. Several other genes have also been studied for obesity-related features. However, these results have been inconsistent [21,22].

Vitamin D and blood pressure

Vitamin D deficiency activates the renin-angiotensin-aldosterone system (RAAS), which increases blood pressure and affects the cardiovascular system. However, randomized clinical trials and meta-analyses have shown conflicting results on the effects of vitamin D supplementation on lowering blood pressure [25,26]. On the other hand, a systematic review and meta-analysis concluded that vitamin D supplementation in patients with vitamin D deficiency who are older than 50 years or obese lowers SBP, while it lowers SBP and DBP in patients with vitamin D deficiency and hypertension [26]. Large, randomized trials with a focus on patients with severe vitamin D deficiency and hypertension are necessary before vitamin D may be recommended for the prevention or treatment of hypertension.

Limitations

This study has several limitations. The significance level of the outcomes could not be attained in our research because of the presumably small sample size. The lack of information on vitamin D dosages is a significant issue because the recommended doses of vitamin D3 may differ substantially. Additionally, dietary data and lipid profiles were unavailable. Therefore, the association between these factors should be investigated in future studies.

Future research should also assess how vitamin D specifically affects the diagnosis, treatment, and prognosis of cardiovascular diseases in children with CKD. Certain issues, such as the cutoff value that would indicate risk and the boundary value that would indicate the necessity for action, still need to be resolved. Additional research is needed to show vitamin D levels as a biomarker for pediatric cardiovascular disorders.

## Conclusions

The results of this retrospective study suggest that children with CKD and lower vitamin D levels have a higher BMI and blood pressure and are therefore at higher risk of developing CVD. Future prospective studies with larger sample sizes are needed to confirm these findings. Additionally, randomized clinical trials are required to determine the effects of sufficient vitamin D status on reducing CVD in patients with CKD.
